# Prevalence and Risk Factors of Retinopathy of Prematurity in Iran

**DOI:** 10.18502/jovr.v14i3.4785

**Published:** 2019-07-18

**Authors:** Mohammad Zarei, Fatemeh Bazvand, Nazanin Ebrahimiadib, Ramak Roohipoor, Reza Karkhaneh, Afsar Farahani Dastjani, Marjan Imani Fouladi, Mohammad Riazi Esfahani, Alireza Khodabande, Samaneh Davoudi, Hamed Ghasemi, Bobeck S Modjtahedi

**Affiliations:** ^1^Eye Research Center, Farabi Eye Hospital, Tehran University of Medical Sciences, Tehran, Iran; ^2^Department of Ophthalmology, Gavin Herbert Eye Institute, University of California, Irvine, CA, USA; ^3^Department of Ophthalmology, University of Florida, Gainesville, Florida, USA; ^4^Eye Monitoring Center, Kaiser Permanente Southern California, CA, USA

**Keywords:** Epidemiology, Frequency, Infants, Pediatric, Retinopathy of Prematurity, Risk Factors

## Abstract

**Purpose:**

The present study aimed to evaluate the frequency and risk factors of retinopathy of prematurity (ROP) among Iranian infants.

**Methods:**

A retrospective cohort study was conducted on infants who had undergone screening for ROP at Farabi Eye Hospital, between March 2016 and March 2017. Data were analyzed based on the presence of extreme prematurity (gestational age ≤ 28 weeks), extremely low-birth-weight (≤ 1000 g), and multiple-gestation (MG) infants.

**Results:**

The prevalence of ROP was 27.28% (n = 543) among all screened infants, 74.4% for extremely preterm (EP) infants, 77.5% for extremely low birth weight (ELBW) babies, and 27.25% for infants from MG pregnancies. On multivariate analysis, gestational age, birth weight, and history of transfusion (P < 0.0001, P < 0.0001, and P = 0.04, respectively) were found to be significantly associated with ROP. More advanced stages of ROP (P < 0.0001) were observed in EP and ELBW infants. Birth weight (P = 0.088), history of transfusion (P = 0.066), and intubation (P = 0.053) were not associated with increased risk of ROP in EP infants, while gestational age (P = 0.037) and history of transfusion (P = 0.040) were significant risk factors for ROP in ELBW infants. Gestational age (P < 0.001) and birth weight (P = 0.001) were significantly associated with ROP in infants from MG pregnancies in multivariate analysis.

**Conclusion:**

ROP remains a commonly encountered disease, especially in ELBW and EP infants. The history of transfusion may have a role in stratifying the risk for ROP and guiding future screening guidelines.

##  INTRODUCTION

Retinopathy of prematurity (ROP) is the leading cause of visual impairment in premature infants and is characterized by abnormal peripheral retinal vascularization.^[1]^ The incidence of ROP is influenced by several factors including gestational age, birth weight, genetics, ethnicity, and level of neonatal care. Gestational age is defined as the length of time of growth of the fetus in the uterus. Screening programs should be regionally tailored to account for these differences. Advances in prenatal and neonatal care have resulted in increasing rates of ROP in the developing world.^[2,3,4]^

Gestational age and birth weight are the two most well-established risk factors for ROP, with other factors such as oxygen therapy, history of transfusion, sepsis, and anemia being less significantly associated with ROP.^[5]^ ROP is diagnosed only in a minority of premature infants, with a smaller subset requiring intervention. As a result, better understanding of risk factors of progression to ROP, especially in those cases requiring treatment, would allow for more effective screening programs.^[5,6]^

The purpose of this study is to evaluate the prevalence of ROP and associated risk factors in a tertiary eye center in Tehran. Additional subgroup analysis was done for patients in three high risk categories: extremely low birth weight (≤ 1,000 g, ELBW), extremely preterm (≤ 28 weeks, EP), and multiple gestational (MG) infants.

##  METHODS

The current retrospective cohort study was performed at Farabi Eye Hospital from March 2016 until March 2017 after obtaining approval from the Institutional Review Board (IRB) of Tehran University of Medical Sciences. The tenets of the Declaration of Helsinki were followed. The medical records of all patients referred to the ROP clinic were assessed. All infants with gestational age ≤ 37 weeks were included in this study. Patients with incomplete medical records were excluded from the analysis. Demographic data (gestational age, birth weight, and gender) and ophthalmic findings (ROP stage, zone, and laterality) were collected and categorized for each patient. Stages of ROP were diagnosed based on the international classification of ROP as follows:

Stage 0: immature retinal vasculature without pathologic changes

Stage 1: presence of a demarcation line between the vascularized and non-vascularized retina

Stage 2: presence of a demarcation line that has height, width, and volume (ridge); small, isolated tufts of neovascular tissue lying on the surface of the retina, commonly known as, “popcorn" may be present.

Stage 3: a ridge with extraretinal fibrovascular proliferation that may be mild, moderate, or severe, as judged by the amount of proliferative tissue present.

Stage 4: partial retinal detachment (A) Extrafoveal retinal detachment; (B) Retinal detachment involving the fovea

Stage 5: total retinal detachment with funnel configuration

Threshold is defined as retinal neovascularization with an extension of either a continuous five clock hour or eight cumulative clock hours in zone I or II with plus disease.

Pre-threshold disease is characterized by all zone I and II changes, except zone II stage 1 and zone II stage 2 without plus disease that do not include the criteria of threshold disease, and is divided into two types:

Type 1:

Zone I, any stage ROP with plus disease,

Zone I, stage 3 ROP without plus disease, or

Zone III, stage 2 or 3 ROP with plus disease

Type 2:

Zone I, stage 1 or 2 ROP without plus disease and

Zone II, stage 3 ROP without plus disease

For the purpose of analysis, the stage of ROP in patients with bilateral disease was categorized based on the eye with the highest stage and lowest zone. Additionally, the history of sepsis, transfusion, acute respiratory distress syndrome (ARDS), oxygen therapy, intubation, intraventricular hemorrhage (IVH), and phototherapy were analyzed as risk factors for ROP. Follow-up visits, indications for treatment, types of treatment, and final ROP status at the last examination were also collected for each patient.

Data was analyzed using IBM SPSS version 19 (Armonk, NY: IBM Corp). Mean ± standard deviation was used to report data. After confirmation of the normal distribution of data by Shapiro-Wilk test, the comparison between birth weight and gestational age of patients with and without ROP was performed using student t-test. Univariate logistic regression was performed to determine the significance of different potential predictive risk factors (gestational age, birth weight, transfusion, sepsis, ARDS, oxygen therapy, intubation, IVH, and phototherapy) in the development of ROP (dependent variable). Subsequently, the resultant significant risk factors were evaluated using multivariate logistic regression to discern the confounding factors. P-value < 0.05 in the t-test and logistic regression was considered statistically significant.

##  RESULTS

###  Subjects

A total of 1,990 patients met the inclusion criteria. Mean gestational age was 32.29 ± 2.75 weeks (range: 24 ± 37 weeks) and mean birth weight was 1,757.99 ± 563.51 g. The cohort comprised of 51.5% (1024) male infants.

###  Retinopathy of Prematurity

The prevalence of ROP was 27.28% (n = 543). Four hundred and sixty-eight patients (23.52%) had bilateral disease and 75 (3.76%) cases had unilateral disease (23 right eyes and 52 left eyes). Stage of ROP is outlined in Table 1. The mean number of examinations and the mean time to the first examination was 2.4 ± 2.6 (1–36) and 7.6 ± 9.1 weeks, respectively. Cases of ROP diagnosed at initial visit were as follows: Pre-threshold 1 (57 patients, 2.9% of the total number of cases), threshold (77 patients, 3.9%), stage 4a (15 patients, 0.75%), stage 5 (26 patients, 1.3%), regressed ROP with unfavorable outcome (3 patients, 0.15%), and other forms of ROP (pre-threshold 2 and less severe) (365 patients, 18.3%). Thirty-two patients were diagnosed with ROP during follow-up visits.

**Table 1:** The rate and stage of ROP in infants with gestational age equal or less than 37 weeks


**ROP**	**Right Eye**	**Left Eye**
	**Frequency (%)**	**Frequency (%)**
No ROP	914 (45.9)	904 (45.4)
Immature retina (avascular area)	585 (29.4)	566 (28.4)
Prethreshold Type 2 and less severe forms of ROP	322 (16.2)	349 (17.5)
Pre-threshold ROP Type 1	60 (3)	56 (2.8)
Threshold ROP	75 (3.8)	76 (3.8)
Stage 4a	11 (0.6)	14 (0.7)
Stage 5	21 (1.1)	22 (1.1)
Autoregressed ROP with unfavorable outcome	2 (0.1)	3 (0.2)
	
	
ROP, retinopathy of prematurity		

###  Risk Factors

Univariate and multivariate logistic regression analyses were performed to examine the relationship between the different risk factors and development of ROP. Gestational age, birth weight, transfusion, ARDS, oxygen therapy, sepsis, intubation, and IVH were significant risk factors on univariate analysis; however, only gestational age, birth weight, and transfusion remained significant on multivariate analysis. [Tables 2 and 3]. Gestational age (P = 0.010), birth weight (P < 0.0001), and IVH (P = 0.028) were significant risk factors for ROP that required treatment.

**Table 2:** Data of univariate regression analysis of different risk factors in the development of retinopathy of prematurity


	**With ROP (** ***n*** ** = 575)**	**Without ROP (** ***n*** ** = 1415)**	**Significance **	**Odds Ratio**	**95% CI**
Gestational age	29.98 ± 2.38	33.34 ± 2.50	< 0.001	0.594	0.565–0.620
Birth weight	1,364.95 ± 417.21	1,936 ± 555.64	< 0.001	0.998	0.997–0.998
Gender (female/male)	292/283	674/741	0.211	0.882	0.724–1.074
Multiple gestations (MGs)	183	458	0.908	0.988	0.799–1.280
Oxygen therapy	379	835	< 0.001	3.320	2.347–4.697
Transfusion	229	190	< 0.001	4.269	3.347–5.444
Intra ventricular hemorrhage	31	27	< 0.001	3.035	1.788–5.149
Sepsis	223	422	0.005	1.358	1.097–1.680
ARDS	391	742	< 0.001	3.320	2.347–4.697
Phototherapy	343	826	0.736	0.955	0.729–1.250
Intubation	58	77	< 0.001	2.146	1.493–3.084
	
	
ARDS, acute respiratory distress syndrome; CI, confidence interval; ROP, retinopathy of prematurity					

**Table 3:** Data of multivariate regression analysis of different risk factors in the development of retinopathy of prematurity


	**Wald **	**Significance **	**Odds Ratio**	**95% CI**
Gestational age	64.002	< 0.001	0.724	0.668–0.783
Birth weight	19.313	< 0.001	0.999	0.999–0.999
Gender	0.807	0.369	0.873	0.650–1.174
Multiple gestations	.011	0.915	1.017	0.747–1.385
Oxygen therapy	0.463	0.496	1.253	0.655–2.397
Transfusion	3.869	0.049	1.355	1.001–1.833
Intra ventricular hemorrhage	2.094	0.148	1.728	0.824–3.624
Sepsis	1.252	0.263	1.172	0.888–1.547
ARDS	1.686	0.194	1.401	0.842–2.333
Phototherapy	0.125	0.724	0.937	0.652–1.345
Intubation	1.480	0.224	1.349	0.833–2.184
	
	
ARDS, acute respiratory distress syndrome; CI, confidence interval				

###  Treatment

One hundred and eighty patients underwent treatment, which included intravitreal injection of bevacizumab [IVB, (155 eyes, 86% of patients)], laser (98 eyes, 54%), scleral buckle (5 eyes, 0.05%), vitrectomy (7 eyes, 4%), combination of IVB and scleral buckle (2 eyes, 1%), and combination of IVB and vitrectomy (1 eye, 0.5%). Twenty-nine patients (47 eyes) required retreatment, including 18 eyes with laser (previous treatment included IVB in 16 eyes and laser in 2 eyes), 17 eyes with IVB (previous treatment included IVB in all), 8 eyes with vitrectomy (previous treatment included laser in 4 eyes and IVB in 4 eyes), and 4 eyes with scleral buckle (previous treatment included laser in 2 eyes and IVB in 2 eyes). Seven eyes required a third treatment: vitrectomy in five eyes (all were primarily treated with IVB, followed by laser), and sclera buckle in two eyes (both received IVB followed by vitrectomy).

###  Extremely Preterm Patients

Two hundred and twenty-three patients were born EP. Mean gestational age and birth weight were 27.24 ± 0.96 weeks and 1,086.50 ± 268.41 g, respectively. Multi-gestation births (56 patients, 25%), transfusion (142 patients, 63.7%), intubation (34 patients, 15.2%), sepsis (102 patients, 46%), ARDS (176 patients, 79%), oxygen therapy (159 patients, 71%, data of oxygen therapy in 34 patients with ARDS were not documented in the archived files), phototherapy (159 patients, 71%), and IVH (20 patients, 9%) were frequently encountered. One hundred and sixty-six (74.4%) EP patients developed ROP: pre-threshold ROP in 32 patients (14%), threshold ROP in 45 patients (20%), stage 4a in 8 patients (3.6%), stage 5 in 12 patients (5.3%), other stages in 68 patients, and unfavorable structural outcome in 1 patient (0.4%). Stage of ROP was more advanced in EP infants than non-EP infants (chi-square test, P < 0.001).

Fifty-seven (25%) EP patients did not develop ROP (no ROP in 20 patients and immature retina in 37 patients). Birth weight, transfusion, and intubation were not significant (although with a trend toward significance) risk factors for ROP in EP infants on multivariate analysis (P = 0.088, 0.066, and 0.053, respectively).

###  Extremely Low Birth Weight Infants

One hundred and sixty patients had ELBW. Transfusion (97 patients, 61%), intubation (25 patients, 15.6%), sepsis (66 patients, 41%), ARDS (123 patients, 77%), oxygen therapy (123 patients, 77%), phototherapy (99 patients, 62%), and IVH (14 patients, 8.7%) were commonly observed. One hundred and twenty-four (77.5%) patients were affected by ROP: 24 (15%) patients with Type 1 pre-threshold ROP, 31 (19%) with threshold ROP, 5 (3%) with stage 4a, 10 (6%) with stage 5, and 54 (33.8%) patients with other stages. Thirty-six patients (22.5%) did not develop ROP (no ROP in 12 patients and immature retina in 24 patients). In multivariate analysis, gestational age (P = 0.037) and transfusion (P = 0.040) were found to be significantly associated with the development of ROP.

###  Multiple Gestations

Six hundred and forty-two babies belonged to MG pregnancies including twins (511 cases, 79.6%), triplets (115 cases, 18%), quadruplets (9 cases, 1.4%), quintuplets (5 cases, 0.8%), and sextuplets (1 case, 0.15%). The mean gestational age was 32.36 ± 2.56 weeks and birth weight was 1,692.94 ± 457.12 g.

Transfusion (130 patients, 20%), intubation (39 patients, 6%), sepsis (216 patients, 33.7%), ARDS (369 patients, 57.4%), oxygen therapy (393 patients, 61.2%), phototherapy (399 patients, 62%), and IVH (16 patients, 2.5%) were encountered. One hundred and seventy-five patients (27.2%) developed ROP: Type 1 pre-threshold ROP in 14 patients (2.2%), threshold ROP in 29 patients (4.5%), stage 4a in 7 patients (1%), stage 5 in 5 patients (0.7%), and other stages in 120 patients (18.7%). Four hundred and sixty-seven patients (72.7%) did not develop ROP, of which 181 (28%) had immature retinas. On multivariate analysis, gestational age and birth weight were shown to be significant risk factors associated with ROP (P < 0.001 and 0.001, respectively). Fifty-eight babies (9%) were treated for ROP. Severity of ROP and the rate of treated cases were similar between MG and single gestation babies.

**Table 4:** The rate of development of ROP and significant risk factors in some studies


**Study **	**Sample size**	**Rate of ROP**	**Risk factors**
Liu et al ${}^{[}{}^{12]}$	472	12.7%	Gestational age, birth weight, preeclampsia, and maternal oxygen supplement before or at the time of delivery
Port et al ${}^{[}{}^{17]}$	582	29%	Univariate analysis: birth weight, gestational age, patent ductus arteriosus, sepsis, intraventricular hemorrhage (IVH), lung disease, days of hyperglycemia, and prescription of postnatal steroids Multivariate analysis: gestational age, days of hyperglycemia, and days of mechanical ventilation
Bossi et al ${}^{[}{}^{18]}$	318	37.1%	Gestational age ≤ 32 weeks, birth weight ≤ 1250 g, oxygen therapy, and sepsis
Kim et al${}^{[}$ ${}^{19]}$	211	42%	Poor weight gain during the first two weeks
This study	1,990	28.9%	Univariate analysis: gestational age, birth weight, transfusion, ARDS, oxygen therapy, sepsis, intubation, and intraventricular hemorrhage (IVH) Multivariate analysis: gestational age, birth weight, and history of transfusion
	
	
ARDS, acute respiratory distress syndrome; ROP, retinopathy of prematurity			

##  DISCUSSION

In this series, the rate of ROP was 27.28% on initial evaluation, with 1.6% developing ROP on subsequent follow-up. The rate of ROP reported in the literature ranges from 11.4 to 44% due to the differences in economic status, ethnicity, genetics, practice setting, screening programs, and level of perinatal care at the respective institutions.^[6,7,8,9,10,11,12,13,14]^ A previous report from Iran found an 8.5% prevalence of ROP among premature infants.^[15]^ The increased rate observed in the current study may be due to improved perinatal care, which has resulted in longer survival of high-risk infants. There is a considerable debate regarding the risk factors for ROP. In the present cohort, low gestational age and birth weight, history of transfusion, ARDS, oxygen therapy, sepsis, intubation, and IVH were significant risk factors for ROP on univariate analysis; however, on multivariate analysis only gestational age, birth weight, and history of transfusion were found to be statistically significant. Gestational age, birth weight, and IVH were significantly associated with the treatment for ROP among all infants on multivariate analysis (P = 0.010, < 0.001 and 0.028, respectively). Table 4 provides a summary of the rates of ROP and risk factors across several studies. Multiple studies have identified lower gestational age and birth weight, higher number of days of oxygen therapy, mechanical ventilation, hyperglycemia, sepsis, transfusion, inadequate weight gain during the first two weeks, and IVH as risk factors for ROP, with low gestational age and birth weight being the most consistently demonstrated risk factors.^[6,8,11,12,16,17,18]^ Khalesi et al.^[19]^ observed gestational age, apnea score in the first minute, oxygen therapy, and phototherapy as significant risk factors, whereas Dai et al.^[5]^ suggested that maternal iron deficiency may be another significant risk factor in the development of ROP. Increased oxygen demand during sepsis as well as toxic effects of high oxygen production during transfusion might result in increased susceptibility to ROP.^[20]^

Similarly, Yau et al.^[21]^ observed that the incidence of ROP in patients with a mean gestational age of 26.4 weeks was 60.7%. Lower birth weight, transfusion, and intubation approached statistical significance as risk factors for ROP in the present cohort of EP patients (P = 0.088, 0.066 and 0.053, respectively). Yau et al^[21]^ retrospectively reviewed EP infants and reported that low gestational age, lighter birth weight, invasive ventilation were significant risk factors for the development of ROP on univariate analysis.

ROP was common in patients with ELBW (77.5%, n = 124), which is consistent with the findings reported by Celebi et al (75.5%).^[25]^ A wide spectrum exists in studies examining the rate of occurrence of ROP (32.8 ± 82.5%) and the rate of treatment for ROP (12.7 ± 48.9%) in ELBW infants.^[4,22,23,24]^ In multivariate analysis, we observed that lower gestational age (P = 0.037) and history of transfusion (P = 0.040) were significant risk factors for the development of ROP in ELBW infants. Celebi et al reported that gestational age, birth weight, history of transfusion, and sepsis were significant factors associated with severe ROP requiring treatment in all infants, while only gestational age was statistically significantly associated with ROP requiring treatment in ELBW infants.^[25]^

Among patients born of MG pregnancies, lower gestational age and birth weight were significant risk factors for ROP (P < 0.001 and 0.001, respectively), and no significant difference existed in the severity of ROP and rate of treatment between MG patients and those born of a single gestation. Twin-twin transfusion syndrome has been found to increase the risk for ROP compared to MG alone (P < 0.01).^[26]^ Yau et al^[27]^ assessed the risk of development of Type 1 ROP in MG infants. MG was found to be a significant risk factor using univariate analysis but not in multivariate analysis. Azad et al^[28]^ observed an increased severity of ROP in heavier MG babies and concluded that weight alone cannot be considered a predictive factor for the severity of ROP. Data from the current study support the view that MG is not a risk factor for the development or severity of ROP.

The current series presents a contemporary analysis of the rate of ROP and risk factors from a large tertiary center in Iran. The strengths of this study include the large sample size and diverse patient population; however, given the retrospective design, confounding and missing data were innate limitations in the analysis of risk factors for ROP. ROP remains a commonly encountered disease with approximately a quarter of preterm infants and three-quarters ELBW or EP infants being affected. Low birth weight and low gestational age are two essential risk factors for the development of ROP. In both developed and developing countries, low gestational age and low birth weight are the commonly reported risk factors for ROP; however, other factors appear to play a larger role in developing countries. On multivariate analysis, transfusion was a significant risk factor for ROP in ELBW infants and approached statistical significance in EP infants. With improvement in perinatal care in developing countries, the burden of ROP is likely to increase. As a result, a better understanding of the patients at risk for ROP, especially among higher risk sub-groups is critical. Future studies should focus on creating models based on multiple risk factors to stratify the risk of ROP and potentially creating more individualized screening guidelines.

##  Financial Support and Sponsorship

Nil.

##  Conflicts of Interest

There are no conflicts of interest.

## References

[1] Casteels I., Cassiman C., Van Calster J., Allegaert K. (2012). Retinopathy of prematurity. *European Journal of Pediatrics*.

[2] Domanico R., Davis D. K., Coleman F., Davis B. O. (2011). Documenting the NICU design dilemma: comparative patient progress in open-ward and single family room units. *Journal of Perinatology*.

[3] Hellström A., Smith L. E. H., Dammann O. (2013). Retinopathy of prematurity. *The Lancet*.

[4] Good W. V., Hardy R. J., Dobson V. (2005). The incidence and course of retinopathy of prematurity: findings from the early treatment for retinopathy of prematurity study. *Pediatrics*.

[5] Dai A. I., Demiryürek S., Aksoy S. N., Perk P., Saygili O., Güngör K. (2015). Maternal Iron Deficiency Anemia as a Risk Factor for the Development of Retinopathy of Prematurity. *Pediatric Neurology*.

[6] Port A. D., Chan R. V., Ostmo S., Choi D., Chiang M. F. (2014). Risk factors for retinopathy of prematurity: insights from outlier infants. *Graefe's Archive for Clinical and Experimental Ophthalmology*.

[7] Good W. V., Hardy R. J., Dobson V. (2005). The incidence and course of retinopathy of prematurity: findings from the early treatment for retinopathy of prematurity study. *Pediatrics*.

[8] Bossi E., Koerner F., Flury B., Zulauf M. (1984). Retinopathy of prematurity: A risk factor analysis with univariate and multivariate statistics. *Helvetica Paediatrica Acta*.

[9] Yin H., HL Li., Zhang W. (2005). Incidence and risk factor analysis of retinopathy of prematurity. Zhonghua Yan Ke Za Zhi. *Li XX*.

[10] Chattopadhyay M. P. A., Pradhan A., Singh R., Datta S. (2015). Incidence and risk factors for retinopathy of prematurity in neonates. *Indian Pediatrics*.

[11] Chen Y., Xun D., Wang Y.-C., Wang B., Geng S.-H., Chen H., Li Y.-T., Li X.-X. (2015). Incidence and risk factors of retinopathy of prematurity in two neonatal intensive care units in North and South China. *Chinese Medical Journal*.

[12] Liu Q., Zin Z., Ke N., Chen L., Chen XK., Fang J. (2014). *Incidence of retinopathy of prematurity in Southwestern China and analysis of risk factors. Med Sci Monit*.

[13] Seiberth V., Linderkamp O. (2000). Risk Factors in Retinopathy of Prematurity. *Ophthalmologica*.

[14] Larsson E. Incidence of ROP in two consecutive Swedish population based studies. *British Journal of Ophthalmology*.

[15] Saeidi R., Hashemzadeh A., Ahmadi S., Rahmani S. (2009). Prevalence and predisposing factors of retinopathy of prematurity in very low-birth-weight infants discharged from NICU. *Iranian Journal of Pediatrics*.

[16] Mohamed S., Murray J. C., Dagle J. M., Colaizy T. (2013). Hyperglycemia as a risk factor for the development of retinopathy of prematurity. *BMC Pediatrics*.

[17] Mutlu F. M., Altinsoy H. I., Mumcuoglu T., Kerimoglu H., Kiliç S., Kul M., Sarici S. Ü., Alpay F. (2008). Screening for Retinopathy of Prematurity in a Tertiary Care Newborn Unit in Turkey: Frequency, Outcomes, and Risk Factor Analysis. *Journal of Pediatric Ophthalmology & Strabismus*.

[18] Kim J., Jin J. Y., Kim S. S. (2015). Postnatal weight gain in the first two weeks as a predicting factor of severe retinopathy of prematurity requiring treatment. *Korean Journal of Pediatrics*.

[19] Khalesi N., Shariat M., Fallahi M., Rostamian G. (2015). Evaluation of risk factors for retinopathy in preterm infant: a case-control study in a referral hospital in Iran. *Minerva Pediatrica*.

[20] Weintraub Z., Carmi N., Elouti H., Rumelt S. (2011). The association between stage 3 or higher retinopathy of prematurity and other disorders of prematurity. *Canadian Journal of Ophthalmology*.

[21] Yau G. S., Lee J. W., Tam V. T., Liu C. C., Wong I. Y. (2014). Risk Factors for Retinopathy of Prematurity in Extremely Preterm Chinese Infants. *Medicine*.

[22] Fortes J. B. F., Eckert G. U., Procianoy L., Barros C. K., Procianoy R. S. (2009). Incidence and risk factors for retinopathy of prematurity in very low and in extremely low birth weight infants in a unit-based approach in southern Brazil. *Eye*.

[23] Kumar P., Sankar M. J., Deorari A., Azad R., Chandra P., Agarwal R., Paul V. (2011). Risk factors for severe retinopathy of prematurity in preterm low birth weight neonates. *The Indian Journal of Pediatrics*.

[24] Fortes Filho J. B., Borges Fortes B. G., Tartarella M. B., Procianoy R. S. (2013). Incidence and Main Risk Factors for Severe Retinopathy of Prematurity in Infants Weighing Less Than 1000 Grams in Brazil. *Journal of Tropical Pediatrics*.

[25] Celebi A. R. C., Petricli I. S., Hekimoglu E., Demirel N., Bas A. Y. (2014). The incidence and risk factors of severe retinopathy of prematurity in extremely low birth weight infants in Turkey. *Medical Science Monitor*.

[26] Gschließer A., Stifter E., Neumayer T., Moser E., Papp A., Dorner G., Schmidt-Erfurth U. (2015). Twin–twin transfusion syndrome as a possible risk factor for the development of retinopathy of prematurity. *Graefe's Archive for Clinical and Experimental Ophthalmology*.

[27] Yau Gordon S. K., Lee Jacky W. Y., Tam Victor T. Y., Yip Stan, Cheng Edith, Liu Catherine C. L., Chu Benjamin C. Y., Yuen Can Y. F. (2014). Differences in Risk Factors for Retinopathy of Prematurity Development in Paired Twins: A Chinese Population Study. *The Scientific World Journal*.

[28] Azad R., Chandra P., Patwardhan S. D., Gupta A. (2010). Profile of asymmetrical retinopathy of prematurity in twins. *Indian Journal of Ophthalmology*.

